# Employing a role playing game and debriefing approach to validate practices and identify variations in response dynamics

**DOI:** 10.1016/j.mex.2018.12.008

**Published:** 2018-12-18

**Authors:** Mandy Doddema

**Affiliations:** Environmental Policy Group, Wageningen University, Netherlands

**Keywords:** Role playing games and debriefing approach, Social practices, Role playing game, Debriefing

## Abstract

To supplement data collected via interviews, participant observation and mapping in two communities where three monitoring interventions had been introduced, a Role Playing Game and debriefing were used to identify variations in fishing and landing practices in proximate communities and to validate responses to three interventions to monitor fishing activity in a tuna fishery in Indonesia. The Role Playing Game was used to simulate the daily routines of fishers and the introduction of three interventions. Debriefing was used to reflect on the internal and external validity of the RPG and to speculate on the impact of the interventions with the players.

• An application of Role Playing Game and debriefing to validate case study findings about changes in social practices resulting from interventions is described.
• Supplementing qualitative research methods with the Role Playing Game and debriefing showcases similarities and variations in social practices and responses to interventions.
• Using the Role Playing Game and debriefing to simulate interventions allows for reflection on implications of interventions with those not (yet) targeted by an intervention.

## Graphical abstract

**Figure:**
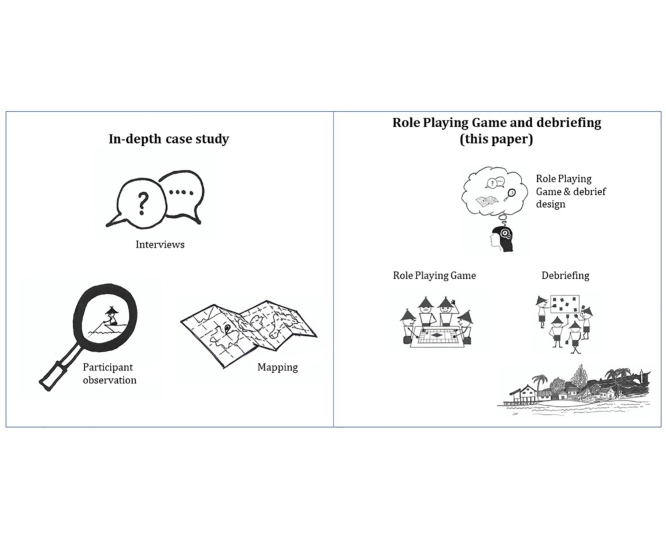

**Specifications Table**

**Table:** 

**Subject area**	Social Sciences
**More specific subject area**	Fisheries sociology
**Method name**	Role playing games and debriefing approach
**Name and reference of original method**	Role playing games: Klabbers [6] Barreteau et al. [10] Debriefing: van den Hoogen et al. [7]
**Resource availability**	n/a

## Method details

This paper showcases how a Role Playing Game (RPG) and debriefing approach was used to supplement an in-depth case study in order to identify and understand the variance of change in social practices in response to interventions designed to monitor fishing activity. The results are presented in Doddema et al. [1].

The researchers employed a social practices perspective to understand the effects of three consecutive monitoring intervention on existing practices. Theories of social practices consider shared and routinized practices as the core units of analysis to understand human behavior and how it changes [[2], [3], [4]]. In the first round of data collection, researchers used a combination of participant observation, in-depth interviews and mapping exercises to zoom in and out on the practices and their configurations [5]. This established how a variety of social practices are performed and linked to each other and how and why monitoring interventions are incorporated, adapted or rejected in existing social practices.

While the findings from the case study offer a detailed understanding of social processes that determine the uptake of interventions, the limited study population and desire to validate findings beyond the two communities led to the development of the RPG and debrief approach.

The reason the RPG and debriefing were chosen to gather additional insights is described now. In constructing the RPG, researchers are able to simulate a ‘real life’ reference system and introduce stimuli that reflect the findings from the first round of data collection. Through game play, researchers can study the player decisions and interactions within the reference system [6]. Following van den Hoogen et al. [7] there is recognition of the challenges of incorporating all contextual components when simulating social practices in the RPG. While RPGs are devices through which players experience a simplified version of reality, the debriefing offers reflection on these experiences which offers a more contextual understanding of the data. Debriefing is a collective assessment of what happened and an evaluation of the relevance of practices outside the RPG. As can be seen in Doddema et al. [1], employing the RPG and debriefing enabled a greater sample of experiences to be collected and assessed, not only in terms of identifying similarities between practices, but also understanding the variance of responses to interventions in a variety of similar but geographically dispersed social settings. The rest of this paper outlines the steps conducted in applying the methodology.

## The Role Playing Game (RPG) and debriefing

Where applicable, we follow the Overview-Design-Details (ODD) protocol [8] to provide a standardized description of the RPG and debriefing.

### Overview

The purpose of research overall is to understand how monitoring interventions are responded to within the context of everyday routines of fishers. The RPG and debriefing were constructed by the researcher to validate the findings from the case study in the first round of data collection and to identify variations in practices and responses.

**Fig. 1:**
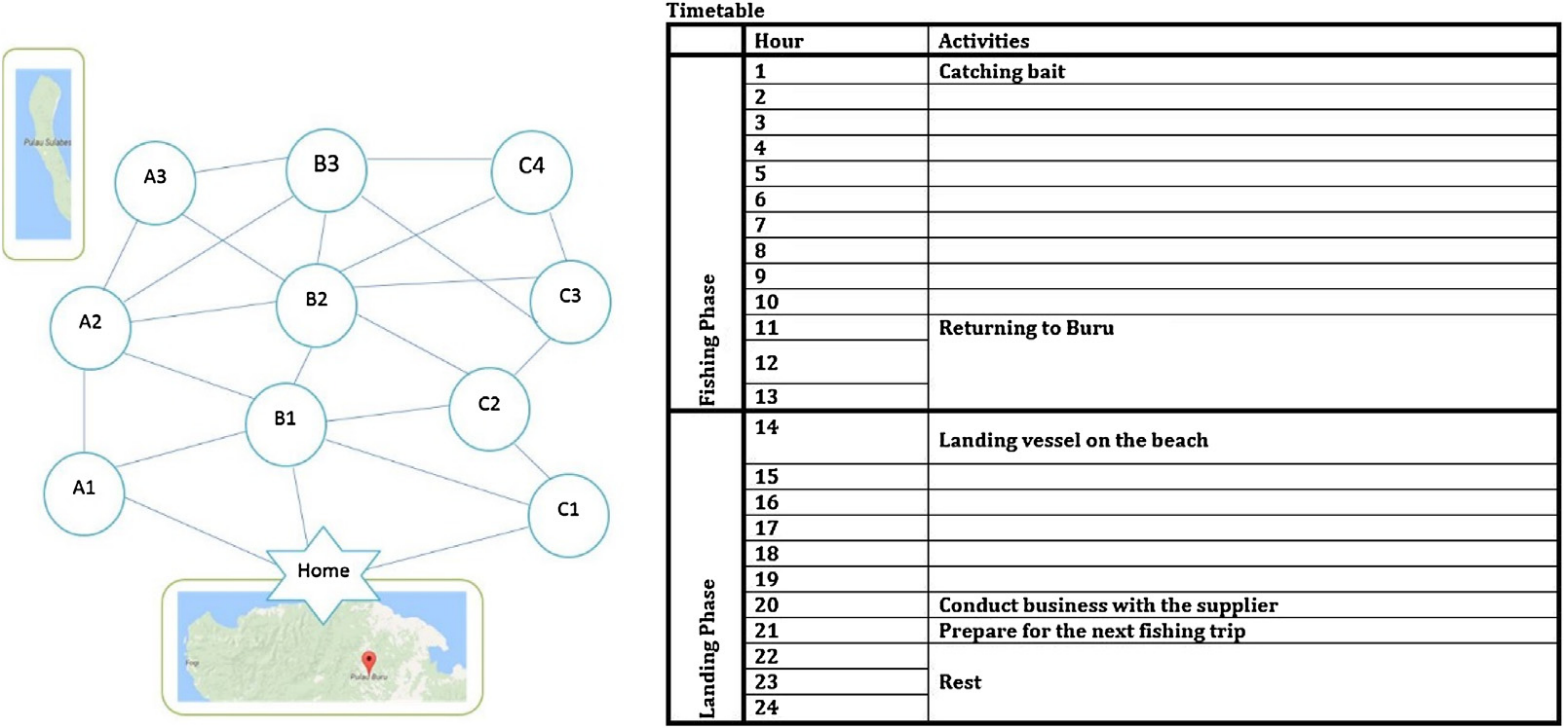
(a) Game board (left)and (b) timetable (right).

In the RPG, four players – who take on the role of handline tuna fishermen - carry out a variety of practices within a fishing day. Players make decisions about how to spend their time in light of interventions in different rounds. Each player sits behind a screen so that other players cannot see their actions. Behind each screen, players have: their personal game board showcasing their home base and 10 fishing areas (circles) and possible movement paths between fishing areas (see Fig. 1a). Each board is populated with dolphin tokens that correspond to the fishing areas indicating the presence or absence of dolphins and a fishing vessel game piece. In addition to the board, each player is provided with an empty timetable (see Fig. 1b), a sticker sheet with all possible actions and the post-fishing activity sheet for that round. Players are urged to act according to their daily routines and to make decisions with regards to the incorporation of the different interventions but players are free to interpret how they do this. The overall goal is that each of the players allocates their time in a way that ensures they catch tuna and also to invest time in various post-fishing trip activities.

The RPG is made up of three rounds and each round represents a fishing day (24 h) which is split into a fishing phase (13 h) and a landing phase (11 h). In the timetable, some hours are already auto-completed. For example, the catching of bait is done in hour 1 so the fishing trip starts in hour 2. Fishers are given a sticker sheet on which all potential moves are indicated and any choice made by the players is logged by the players by sticking the stickers in the relevant hour slot in the timetable.

#### Playing the RPG

Each round is made up of three phases:

Fishing phase: In each round, there is one dolphin token that indicates that dolphins (and thus tuna) are present, all other fishing areas contain dolphin tokens that indicate dolphins are absent. During this phase the players have to search for the fishing area where the dolphins are present. Once a player has found the dolphins, they can start fishing for the tuna.

a Searching for tuna: Players search for tuna by moving their fishing vessel token on the game board to different fishing areas. Once the fishing vessel is moved to a new fishing area, this is documented in the timetable by putting the sticker with the fishing area name (e.g. A1) on the associated hour slot in the timetable. The player then checks whether there is dolphin present/absent on the flipside of the dolphin token. If there is no dolphin present, the player removes the dolphin token from the game board and continue searching for the dolphin pod. If there is a dolphin pod present the player can start fishing (see b).
  • Every move takes one hour.
  • There is only one fishing area that has dolphins in each round.
  • Players have to stop when they reach a new fishing area. For example, a player can’t move their fishing vessel from A1 to A3, they also have to stop at A2.
  • Players can only move their fishing vessels to new fishing areas that are connected via lines to the previous fishing area.
b Fishing: Once a player finds the dolphins, they throw the die to determine whether they catch tuna and if so, how long it takes to bring in the tuna (see Table 1). The die results are noted in the timetable by sticking the catch or no catch sticker and on the associated hour slot in the timetable.

**Table 1:** Die results.

Die result	# of tuna caught?	Time needed to haul in tuna	Description
1 or 2	1 tuna	1 h	a player catches 1 tuna and that is takes them 1 h to bring the tuna on board and loin the tuna
3 or 4	1 tuna	2 h	a player catches 1 tuna and that is takes them 2 h to bring the tuna on board and loin the tuna. When this occurs a player has to wait an extra move before they can carry out the next move.
5 or 6	0 tuna	n/a	No tuna is caught

Landing phase: During this phase, players return home from the fishing trip. Aside from some auto-completed activities in the timetable the players have to choose how to spend the remaining hours in the timetable using the post-fishing activities sheet. Using numbered stickers that correspond to the post fishing activities they indicate their choices by putting a sticker with the corresponding number in the timetable. Each round, players can choose between eight activities (these change each round). Players are free to choose how many hours their spend on each activity so they could choose to do five different activities or three different activities of which two are done for two hours or even to do only one of the activities for five hours. Players make their choice and put all the stickers in the timetable at the same time. In round 2 and 3, additional information is provided before players complete the timetable.

Reflection on the day: After all players have completed the timetable they reflect on their trip and post-fishing activities for 5 min with the game master. This is a short intermezzo so that the next round can be set up.

#### Interventions introduced per round

Per round, the introduction of the interventions takes place before the fishing phase starts or before the landing phase starts:

*Round 1:* At the start of round 1, the game master tells the players that they aren’t allowed to communicate with other players in this round. During the landing phase in round 1, the game master invites “the NGO representative” to introduce the data collection program to the players. Data collection is a post-fishing activity which takes 1 h which gives fishers an overview of their catches.

*Round 2:* At the start of round 2, fishers are split up into 2 fishers groups (2 players in each) and are given SMS notes to communicate during the fishing phase. At any point during the fishing phase, players can pass a SMS note to another player to give or ask information about where the tuna is located. The game master explains that the fishers join the fishers group because their middlemen encouraged them to do this. Being part of the group means that a percentage of the income of all the fishers in the group is given back to the group to spend on community projects. The income is awarded based on how much is caught per group. During the landing phase in round 2, the NGO representative comes to the fishers to tell them about the new post-fishing activity – completing the self-reporting form which is connected to the group income but it is not mandatory which is a new post-fishing activity. It takes two hours to fill out the form because it takes fishers a while to learn how to fill out the form.

*Round 3:* At the start of round 3, the NGO representative explains that they have stopped the self-reporting form because they realized it was taking a lot of time from the fishers. As an alternative to collect the data they are now introducing a vessel tracker which is attached to the front of the boat. The vessel tracker lets the NGO representative know exactly where the fishers are fishing but does not give the fishers any information during their fishing trips. Because the NGO knows where the fishers are fishing, they can also locate them if the weather is bad so there is a safety element to bringing it. Choosing to bring the vessel tracker doesn’t cost the players any time. Players are given a note on which they indicate whether they want to bring the vessel tracker or not. During the landing phase in round 3, the NGO representative comes to thank them for using the vessel tracker.

### Design concepts

The RPG was developed in line with theories of social practices [[2], [3], [4]] and based on the case study in two communities confronted with the monitoring interventions.

The construction of this RPG was loosely based on the Actor-Resources-Dynamics-Interactions (ARDI) method. This method identifies the stakeholders concerned with the key research question, the management entities, the resources used and the main processes driving change [9]. The case study identified that the established daily routines and practices were important for how fishers responded to the interventions. As such, in designing the RPG the goal was to create a simplified representation of daily routines and how the interventions land in the practices i.e. how they are understood and accepted by all players as plausible. Given the temporal and spatial constraints of the practices, the format of the RPG was a conceptualization of a fishing day. The RPG was tested and updated according to feedback from players in three sessions, one with the NGO and two sessions with fishers.

The dynamics of where the tuna is and the probability of catching the tuna were based on mapping exercises carried out with fishers in case study. The post-fishing activities were identified based on interviews and participant observation in the case study. The NGO representative’s pitch to the fishers at various points in the RPG was also based on NGO narratives identified in case study.

### Details (and data collection)

The RPG follows a strict protocol of play starting with a 10-minute introduction and explanation of the materials and phases of the game. The RPG unfolds for one hour and is followed up by a debriefing session of one hour. The RPG is facilitated by a Game Master who is in charge of introducing the RPG, controlling time and having short reflection rounds with the players in between each round. Other tasks covered by the facilitating team include taking on the role of the ‘NGO’ in round two and three, collecting players timetables, resetting the RPG at the start of each round and keeping track of the nature and interactions between actors.

At the start of each round, players are provided with a timetable of the day on which they record all their actions. In the fishing phase this is about decisions of where to search for tuna and once found, documenting whether or not they catch tuna. In the landing phase, this is about the decisions of which post-fishing activities to do upon returning from the fishing trip. During the course of the RPG, the facilitating team keeps track of which players choose which interventions. Furthermore, social interactions between players are tracked by the facilitation team to inform the debriefing and all sessions are recorded on video and audio with prior informed consent of all players.

After all the rounds are played, the players together with the game master debrief the different aspects of the RPG following the framework developed by van den Hoogen [7]. The debriefing sessions are audio recorded with prior informed consent of all players and transcribed and analysed using content analysis in atlas.ti. The following questions structured the debrief sessions, though more in depth questions were asked based on answers.

1 What did you think about playing the game?
2 What happened in the game?
3 Which of you choose to do the enumeration? Why did you (not) choose to do the enumeration?
4 Which of you choose to do the self-reporting? Why did you (not) choose to do the self-reporting?
5 Which of you choose to bring the Spot Trace? Why did you (not) choose to do the Spot Trace?
6 How did the introduction of the fisher groups change the fishing phase? And the landing phase?
7 Did the introduction of the fisher groups change the relationships with the NGO? Other fishers? The middleman?
8 How does the fishing phase reflect what you do on a fishing trip?
9 How does the landing phase reflect what you do after returning from a fishing trip?
10 What things are missing from the game if trying to understand how fishers to choose to do specific activities?
11 In the game, fishers receive a group income for being part of fisher groups and meeting requirements. In reality, how do fishers in the community interact?
12 In the game, the middleman plays a small role. In reality what is the role of middlemen in everyday life in the community?
13 If these interventions were introduced in your community what do you think you would do and what other factors influence it? What things would enable or constrain fishers in your community to adopt or reject the different interventions?
14 In the game, fisher groups are formed around different suppliers. In reality, if groups were formed around suppliers, how do you think that this would change the relationship between fishers?
15 In the game, doing enumeration, self-reporting and Spot Trace requires time but also has benefits. How do you think an NGO would be received by the fishers in your community? What kinds of things would an NGO need to be sensitive to if they were to want to work with the fishers in your community?

Within two days of RPG and debrief, the team organized one-to-one interviews with each of the players. The interviews we similar to the semi-structured interviews conducted in the case study (see Appendix A in [1]) and focused on understanding the existing practices in these communities as well as the similarities and differences with those in the case study communities.

## Concluding remarks

The goal of this paper was to showcase how RPGs and debriefing were used to supplement results from an in-depth case study on the social processes of intervention uptake. While the initial round of data collection from the case study offers in-depth insights, the RPG and debriefing together allow for expanding the study population to include insights from fishing communities that were not targeted by the interventions. It is the combination of the case study and the RPG and debrief that enabled in-depth understanding of a specific case but also to have broader reflections on the implication of the interventions in similar settings which were key for generating the extensive results for the overall study.
